# Faces in Context: A Review and Systematization of Contextual Influences on Affective Face Processing

**DOI:** 10.3389/fpsyg.2012.00471

**Published:** 2012-11-02

**Authors:** Matthias J. Wieser, Tobias Brosch

**Affiliations:** ^1^Department of Psychology, University of WürzburgWürzburg, Germany; ^2^Department of Psychology, University of GenevaGeneva, Switzerland

**Keywords:** facial expression, face perception, emotion, context, “basic emotion”

## Abstract

Facial expressions are of eminent importance for social interaction as they convey information about other individuals’ emotions and social intentions. According to the predominant “basic emotion” approach, the perception of emotion in faces is based on the rapid, automatic categorization of prototypical, universal expressions. Consequently, the perception of facial expressions has typically been investigated using isolated, de-contextualized, static pictures of facial expressions that maximize the distinction between categories. However, in everyday life, an individual’s face is not perceived in isolation, but almost always appears within a situational context, which may arise from other people, the physical environment surrounding the face, as well as multichannel information from the sender. Furthermore, situational context may be provided by the perceiver, including already present social information gained from affective learning and implicit processing biases such as race bias. Thus, the perception of facial expressions is presumably always influenced by contextual variables. In this comprehensive review, we aim at (1) systematizing the contextual variables that may influence the perception of facial expressions and (2) summarizing experimental paradigms and findings that have been used to investigate these influences. The studies reviewed here demonstrate that perception and neural processing of facial expressions are substantially modified by contextual information, including verbal, visual, and auditory information presented together with the face as well as knowledge or processing biases already present in the observer. These findings further challenge the assumption of automatic, hardwired categorical emotion extraction mechanisms predicted by basic emotion theories. Taking into account a recent model on face processing, we discuss where and when these different contextual influences may take place, thus outlining potential avenues in future research.

## Introduction

Dating back to Darwin (1872) it has been proposed that emotions are universal biological states that are accompanied by distinct facial expressions (Ekman, 1992). This “basic emotion” approach assumes that the expressions of emotion in a face and their perception are unique, natural, and intrinsic phenomena (Smith et al., 2005) that are reliable markers of emotions, co-vary with the subjective experience, belong to a whole set of emotional responses, are readily judged as discrete categories, and as such are essential for successful and efficient social interaction (Matsumoto et al., 2008).

As a consequence, experimental work on the perception of emotional facial expressions has often relied on a set of isolated, de-contextualized, static photographs of actors posing facial expressions that maximize the distinction between categories (Barrett et al., 2011) and give researchers substantial control of the duration, appearance, and physical properties of the stimulus. This work has demonstrated that humans generally are able to identify facial expressions with high accuracy when these are presented as singletons (Matsumoto, 2001; Elfenbein and Ambady, 2002). With respect to the underlying neural mechanisms, cognitive neuroscience research has revealed that emotional face processing engages a widely distributed network of brain areas (Haxby et al., 2000; Haxby and Gobbini, 2011). Core brain regions of face processing are located in inferior occipital gyrus (occipital face area, OFA, Puce et al., 1996), lateral fusiform gyrus (fusiform face area, FFA, Kanwisher et al., 1997), and posterior superior temporal sulcus (pSTS, Hoffman and Haxby, 2000). According to an influential model of face perception, incoming visual information is first encoded structurally, based on the immediate perceptual input, and then transformed into a more abstract, perspective-independent model of the face that can be compared to other faces in memory (Bruce and Young, 1986). The structural encoding phase has been linked to computations in OFA, whereas the more abstract, identity-based encoding occurs in FFA (Rotshtein et al., 2005). The pSTS has been linked to the processing of dynamic information about faces including social signals such as eye gaze and emotional expression (Haxby and Gobbini, 2011). Other regions involved in face processing are amygdala and insula, implicated in the processing of emotion and facial expressions, regions of the reward circuitry such as caudate and orbitofrontal cortex, implicated in the processing of facial beauty and sexual relevance, and inferior frontal gyrus, implicated in semantic aspects of face processing and medial prefrontal cortex, implicated in theory of mind operations when viewing familiar faces of relatives and friends (Haxby et al., 2000; Ishai, 2008).

As mentioned above, the neurocognitive model of facial expression processing has been developed based on experiments using single, context-less faces, a situation rather artificial and low in ecological validity^1^. Outside the laboratory, however, faces rarely are perceived as single entities and most likely appear within a situational context, which may have a strong impact on how they are perceived.

**Figure 1:**
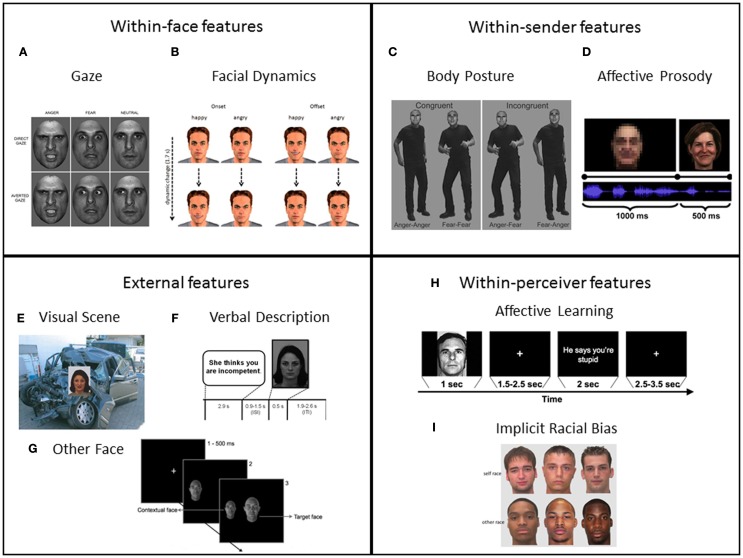
**Examples of the research paradigms established to investigate contextual influences on affective face processing along the nomenclature of within-face, within-sender, external, and within-perceiver features: (A)** Eye gaze: direct versus averted eye gaze combined with angry, fearful, and neutral facial expressions. Reproduced with permission from Ewbank et al., 2010. **(B)** Facial dynamics: time course (start and end frame of video clips) of facial expressions (happy versus angry), which were used to investigate onset versus offset of facial expressions. Reproduced with permission from Mühlberger et al., 2011. **(C)** Compound facial expressions + body postures (congruent and incongruent, anger and fear is expressed in faces and body postures). Reproduced with permission from Meeren et al., 2005. **(D)** Concurrent affective prosody presented simultaneously with (happy) facial expression (after blurred baseline condition). Adapted with permission from Müller et al., 2011. **(E)** Visual affective background picture (negative, threat) presented together with facial expression (fear). Adapted with permission from Righart and de Gelder, 2008a. **(F)** Preceding verbal description (negative) used as situational context for neutral face. Adapted with permission from Schwarz et al., 2012. **(G)** Task-irrelevant context faces presented together with target face (center of the screen). Adapted with permission from Mumenthaler and Sander, 2012. **(H)** CS + trial (with social UCS consisting of verbal insult) used in a social conditioning paradigm. Reproduced with permission from Davis et al., 2010. **(I)** Self race (here White) versus other race (here Black) faces as typically used in studies on implicit racial bias. Adapted with permission from Lucas et al., 2011.

In this article, we review recent studies in which contextual influences on facial expression perception were investigated at the level of subjective perception and at the level of the underlying neural mechanisms. According to Brunswik’s functional lens model of perception (Brunswik, 1956; see also Scherer, 2003), the transmission of a percept (such as an emotional expression) can be broken down into different stages, including the encoding of a state into distal cues by the sender, the actual transmission of the cues, and the perception of the proximal cues by the receiver. Accordingly, we will structure our review following this sequence, beginning with contextual effects occurring mainly during the encoding of the expression, followed by effects occurring during the transmission from sender to receiver, and finally looking at effects occurring mainly during the decoding/perception by the receiver. We additionally introduce a distinction between distal context effects coming from the face of the sender and effects transmitted by other channels of the sender, which results in the following four types of contextual influences on face perception: (1) within-face features (i.e., characteristics that occur in the same face as the “core” facial expression such as eye gaze and facial dynamics), (2) within-sender features (i.e., concurrent context cues from the sender such as affective prosody and body posture), (3) external features from the environment surrounding the face (i.e., concurrent multisensory cues originating outside the sender such as visual scene, other faces, social situations), and (4) within-perceiver features (i.e., affective learning processes and implicit processing biases in the perceiver). Following this systematization of different types of context, we review and summarize experimental findings from studies where these four types of features served as context cues for the perception of facial expressions (see Figure 1).

## Contextual Influences on Face Recognition and Perception

### Within-face features

#### Eye gaze

Within the face, eye gaze is probably the most powerful contextual cue. For example, expressions of joy and anger appear to be considerably more intense when combined with direct than with averted gaze (Adams and Kleck, 2003, 2005). In a series of studies it has been shown that the categorization of happy and sad expressions as well as angry and fearful expressions was impaired when eye gaze was averted, in comparison to direct gaze conditions (Bindemann et al., 2008). However, other studies suggest an interaction of gaze direction and facial expressions, in that angry faces appear more intense and are more easily recognized when paired with a direct gaze, whereas the opposite seems to be true for fearful faces (Adams et al., 2003, 2006; Adams and Kleck, 2005; Sander et al., 2007; Adams and Franklin, 2009; Benton, 2010; Ewbank et al., 2010). This has been explained in the context of appraisal theory of emotion, which assumes that gaze and expression are always integrated by the observer during an appraisal of stimulus relevance (for a recent review, Graham and Labar, 2012). In line with this assumption, angry faces are evaluated as being angrier when showing direct gaze, as eye contact implies a potential threat in form of imminent attack of the sender, while fearful faces are perceived as more fearful when showing averted gaze, as this might indicate potential threat in the environment (Adams and Kleck, 2003; Sander et al., 2007). These behavioral results were replicated by N’Diaye et al. (2009) who additionally observed increased activity in the amygdala as well as in fusiform and medial prefrontal areas to angry faces with direct gaze and fearful faces with averted gaze. This effect was found to be absent or considerably reduced in patients with right and left amygdala damage (Cristinzio et al., 2010). A recent study showed that amygdala responses to rapidly presented fear expressions are preferentially tuned to averted gaze, whereas more sustained presentations lead to preferential responses to fearful expressions with direct gaze (Adams et al., 2012a). This is in line with findings showing that gaze direction modulates expression processing only when the facial emotion is difficult to discriminate (Graham and LaBar, 2007). Moreover, it has been demonstrated that when gaze direction is rendered ambiguous and embedded in explicit, contextual information about intentions of angry and fearful faces, a similar pattern of amygdala activation is observed as in prior results to non-ambiguous gaze cues: angry faces, which were contextualized by explicit information (“The people bumped into you by accident. You are in a bad mood today and so you start to insult them. Thereupon, the people become very angry at/afraid of you”) targeting the observer elicited stronger amygdala responses than angry faces targeting another person, whereas the opposite pattern was observed for fearful faces (Boll et al., 2011). By showing that contextual information interacts with facial expression in the same manner as gaze direction, this study underlines the significance and meaning of certain gaze directions for human observers. Altogether, gaze interacts with facial expression, but this also depends on the relative timing and the nature of the stimuli used. The neural substrates seem to mainly involve the STS and the amygdala, suggesting that the amygdala is involved in processes going beyond basic emotion recognition or arousal processing, as integral part of an appraisal system that is sensitive to expression and gaze direction, among other features (Graham and Labar, 2012).

#### Facial dynamics

The temporal dynamics of facial movements are a further contextual cue expressed in the face. Particularly the time course of facial movements when expressing an emotion as well as whether an expression starts developing (i.e., a face turns from neutral to angry) or ends (i.e., a face turns neutral from angry) may constitute important non-emotional context cues. It has been demonstrated several times that dynamically evolving facial expressions are much better recognized, rated as more arousing, elicit larger emotion-congruent facial muscle responses (Weyers et al., 2006; Sato and Yoshikawa, 2007a,b), and also elicit stronger amygdala activity compared to static faces (LaBar et al., 2003; Sato et al., 2004). Comparing dynamic to static faces, enhanced emotion-specific brain activation patterns have been found in the parahippocampal gyrus including the amygdala, fusiform gyrus, superior temporal gyrus, inferior frontal gyrus, and occipital and orbitofrontal cortex (Trautmann et al., 2009). Moreover, dynamic compared to static facial expressions are associated with enhanced emotion discrimination at early and late stages of electro-cortical face processing (Recio et al., 2011). Comparing onset and offset of facial expressions, it has been shown that perceived valence and threat of angry and happy facial expressions depend on their dynamics in terms of on-versus offset of the respective facial expression. While the onset of happy facial expressions was rated as highly positive and not threatening, the onset of angry facial expressions was rated as highly negative and highly threatening. Furthermore, the offset of angry compared to the onset of angry facial expressions was associated with activity in reward-related brain areas, whereas onset of angry as well as offset of happy facial expressions were associated with activations in threat-related brain areas (Mühlberger et al., 2011).

### Within-sender features

#### Affective prosody

Besides concurrent visual information, acoustic information from the sender may also serve as context for the perception, recognition, and evaluation of facial expressions. Indeed, the identification of the emotion in a face is biased in the direction of simultaneously presented affective prosody (de Gelder and Vroomen, 2000). Furthermore, it was demonstrated that this effect occurred even under instructions to base the judgments exclusively on information in the face. Increased accuracy rates and shorter response latencies in emotion recognition tasks when facial expressions are combined with affective prosody have been found in several other studies (de Gelder et al., 1999; Dolan et al., 2001). In one of the first neuroimaging studies on face and affective prosody processing, Dolan et al. (2001) contrasted emotional congruent to emotional incongruent conditions in an audiovisual paradigm and found greater activation of the left amygdala and right fusiform gyrus in congruent as compared to incongruent conditions. Further fMRI studies on the audiovisual integration of non-verbal emotional information revealed that this perceptual gain during audiovisual compared to visual stimulation alone is accompanied by enhanced BOLD responses in pSTG, left middle temporal gyrus and thalamus when comparing the bi-modal condition to either unimodal condition (Pourtois et al., 2005; Ethofer et al., 2006; Kreifelts et al., 2007). In a recent neuroimaging study it has been found that subjects rated fearful and neutral faces as being more fearful when accompanied by sounds of human screams than compared to neutral sounds (Müller et al., 2011). Moreover, the imaging data revealed that an incongruence of emotional valence between faces and sounds led to increased activation in areas involved in conflict monitoring such as the middle cingulate cortex and the right superior frontal cortex. Further analyses showed that, independent of emotional valence congruency, the left amygdala was consistently activated when the information from both modalities was emotional. If a neutral stimulus was present in one modality and emotional in the other, activation in the left amygdala was significantly attenuated compared to when an emotional stimulus was present in both modalities. This result points at additive effects in the amygdala when emotional information is present in both modalities. This congruency effect was also recently confirmed in a study where laughter as played via headphones increased the perceived intensity of a concurrently shown happy facial expression (Sherman et al., 2012). Taken together, results from audiovisual paradigms point at massive influences of auditory context cues in terms of affective prosody on the processing of facial expressions. Again, this is reflected by congruency effects with better recognition rates and larger activations in face processing and emotion processing areas.

#### Body postures

The most obvious within-sender context for a face is the body it belongs to, which also underlies non-verbal communication via postures and other body language (e.g., gestures). Meeren et al. (2005) found that observers judging a facial expression are strongly influenced by the concomitant emotional body language. In this study, pictures of fearful and angry faces and bodies were used to create face-body compound images, with either congruent or incongruent emotional expressions. When face and body conveyed conflicting emotional information, the recognition of the facial expression was attenuated. Also, the electro-cortical P1 component was enhanced in response to congruent face-body compounds, which points to the existence of a rapid neural mechanism assessing the degree of agreement between simultaneously presented facial and bodily emotional expressions already after 100 ms. Van den Stock et al. (2007) used facial expressions morphed on a continuum between happy and fearful, and combined these with happy or fearful body expressions. Ratings of facial expressions were influenced by body context (congruency) with the highest impact for the most ambiguous facial expressions. In another study, it was shown that identical facial expressions convey strikingly different emotions depending on the affective body postures in which they were embedded (Aviezer et al., 2008). Moreover, it was shown that eye movements, i.e., characteristic fixation patterns previously thought to be determined solely by the facial expression, were systematically modulated by this emotional context. These effects were even observed when participants were told to avoid using the context or were led to believe that the context was irrelevant (Aviezer et al., 2011). In addition, these effects were not influenced by working memory load. Overall, these results suggest that facial expressions and their body contexts are integrated automatically, with modulating effects on perception in both directions.

### External features

#### Emotion labels

Emotion research frequently uses language in form of emotion labels, both to instruct participants and to assess recognition performance. As the following examples demonstrate, language can guide and even bias participants on how to read facial expressions in this kind of research. For example, when participants were asked to repeat an emotion word such as “anger” aloud either three times (temporarily increasing its accessibility) or 30 times (temporarily reducing its accessibility), reduced accessibility of the meaning of the word led to slower and less accurate emotion recognition, even when participants were not required to verbally label the target faces (Lindquist et al., 2006). In a similar vein, morphed faces depicting an equal blend of happiness and anger were found to be perceived as angrier when those faces were paired with the word “angry” (Halberstadt and Niedenthal, 2001). It has furthermore been demonstrated that verbalizing words disrupts the ability to make correct perceptual judgments about faces, presumably because it interferes with access to language necessary for judgment (Roberson and Davidoff, 2000). In line with the latter findings, at the neural level it was demonstrated that emotional labeling of negative emotional faces produced a decreased response in limbic brain areas such as the amygdala, which might reflect that putting feeling into words has regulatory effects at least for negative emotions (Lieberman et al., 2007). Overall, these findings are in line with the language-as-a-context-hypothesis (Barrett et al., 2007), which proposes that language actively influences emotional perception by dynamically guiding the perceiver’s processing of structural information from the face.

#### Verbal descriptions of social situations

Verbal descriptions of social situations also provide strong contextual cues which influence facial expression perception. Carroll and Russell (1996) let participants read six short stories of situations which created a context, afterward a facial expressions was shown and had to be rated. Indeed, in each of the 22 cases that were examined, contextual information overwrote the facial information (e.g., a person in a painful situation but displaying fear was judged as being in pain). These results are in line with an earlier study, where verbal descriptions of emotion-eliciting events were used as situational cues, which increased emotion recognition in faces (Trope, 1986). Using neuroimaging it was demonstrated that brain responses to ambiguous emotional faces (surprise) are modified by verbal descriptions of context conditions: stronger amygdala activation for surprised faces embedded in negative compared to positive contexts was found, thus demonstrating context-dependent neural processing of the very same emotional face (Kim et al., 2004). This effect has been recently extended to neutral faces, where context conditions of self-reference modulated perception and evaluation of neutral faces in terms of larger mPFC and fusiform gyrus activity to self- versus other-related neutral faces and more positive and negative ratings of the neutral faces when put in positive and negative contexts (Schwarz et al., 2012). The latter findings demonstrate that contextual influences might be most powerful when the information about the emotion from facial features is absent or ambiguous at best.

#### Other faces

Probably the most frequent external context cue for a face is another face, as we often perceive persons surrounded by persons. Not surprisingly, it was demonstrated that facial expressions have a strong influence on the perception of other facial expressions. Russell and Fehr (1987) observed that the read-out of an emotion from a facial expression clearly depends on previously encountered facial expressions: a first expression (the anchor) displaced the judgment of subsequent facial expression, for instance, a neutral target face was categorized as sadder after a happy face was seen. In a series of three experiments it was shown that the implicit contextual information consisting of other facial expressions modulates valence assessments of surprised faces, such that they were interpreted as congruent with the valence of the contextual expressions (Neta et al., 2011). Recently, it was also demonstrated that congruent, but irrelevant faces in the periphery enhance recognition of target faces, whereas incongruent distracter faces reduced recognition of target faces (Mumenthaler and Sander, 2012). This contextual effect of concurrent faces was augmented when the peripheral face gazed at the target face, indicating social appraisal, where the appraisal of another person is integrated into the appraisal of the observer, thus facilitating emotion recognition. Social appraisal was furthermore demonstrated by facilitated recognition of fear in a centrally presented face when an angry peripheral face gazed at the central face. In addition to emotion recognition of the target, emotional expressions of context faces may also be used to inform the explicit social evaluations of the observer: male faces that were looked at by females with smiling faces were rated as more attractive by female participants than males looked at with neutral expressions. Revealing an interesting gender difference, in the same experiment male participants preferred the male faces that were being looked at by female faces with neutral expressions (Jones et al., 2007).

#### Visual scene

Faces usually are perceived together with other visual stimuli, for example the surrounding visual scene which might be constituted by non-animated visual objects. Barrett and Kensinger (2010) found that visual context is routinely encoded when facial expressions are observed. Participants remembered the context more often when asked to label an emotion in a facial expression than when asked to judge the expression’s simple affective significance (which can be done on the basis of the structural features of the face alone). The authors conclude that the structural features of the face, when viewed in isolation, might be insufficient for perceiving emotion. In a series of studies, Righart and de Gelder, 2006, 2008a,b) examined how the surrounding visual context affects facial expression recognition and its neural processing. Using event-related brain potentials to faces (fearful/neutral) embedded in visual scene contexts (fearful/neutral) while participants performed an orientation-decision task (face upright or inverted), they found that the presence of a face in a fearful context was associated with enhanced N170 amplitudes, and this effect was strongest for fearful faces on left-occipito-temporal sites (Righart and de Gelder, 2006). Interestingly, faces without any context showed the largest N170 amplitudes, indicating competition effects between scenes and faces. In a follow-up study, participants had to categorize facial expressions (disgust, fear, happiness) embedded in visual scenes with either congruent or incongruent emotional content (Righart and de Gelder, 2008b). A clear congruency effect was found such that categorization of facial expressions was speeded up by congruent scenes, and this advantage was not impaired by increasing task load. In another study, they investigated how the early stages of face processing are affected by emotional scenes when explicit categorizations of fearful and happy facial expressions are made (Righart and de Gelder, 2008a). Again, emotion effects were found with larger N170 amplitudes for faces in fearful scenes as compared to faces in happy and neutral scenes. Critically, N170 amplitudes were significantly increased for fearful faces in fearful scenes as compared to fearful faces in happy scenes and expressed in left-occipito-temporal scalp topography differences. Using videos as visual context, it was also demonstrated that both positive and negative contexts resulted in significantly different ratings of faces compared with those presented in neutral contexts (Mobbs et al., 2006). These effects were accompanied by alterations in several brain regions including the bilateral temporal pole, STS insula, and ACC. Moreover, an interaction was observed in the right amygdala when subtle happy and fear faces were juxtaposed with positive and negative movies, respectively, which again points at additive effects of context and facial expression. Together, these series of experiments clearly indicate that the information provided by the facial expression is automatically combined with the scene context during face processing. Mostly, congruency effects were observed such that congruent visual contexts helped identifying facial expressions and led to larger N170 amplitudes^2^ and alterations in face processing areas in the brain.

### Within-perceiver features

#### Social affective learning mechanisms and recognition memory

When we process a face, we compare it to previously encoded memory representations. Affective information stemming from previous encounters may thus guide our perception and evaluation. Affective or social conditioning studies have investigated this effect by pairing neutral faces with social cues (e.g., affective sounds, sentences) serving as unconditioned stimulus (UCS). For example, in one study, participants learned that faces predicted negative social outcomes (i.e., insults), positive social outcomes (i.e., compliments), or neutral social outcomes (Davis et al., 2010). Afterward, participants reported liking or disliking the faces in accordance with their learned social value. During acquisition, differential activation across the amygdaloid complex was observed. A region of the medial ventral amygdala and a region of the dorsal amygdala/substantia innominata showed signal increases to both negative and positive faces, whereas a lateral ventral region displayed a linear representation of the valence of faces such that activations in response to negatively associated faces were larger than those to positive ones, which in turn were larger compared to those elicited by faces associated with neutral sentences. In another social conditioning paradigm, Iidaka et al. (2010) found that a neutral face could be negatively conditioned by using a voice with negative emotional valence (male voice loudly saying “Stupid!”). Successful conditioning was indicated by elevated skin conductance responses as well as greater amygdala activation, demonstrating that the “perceptually” neutral face elicited different behavioral and neural responses after affective learning. Moreover, Morel et al. (2012) showed in a MEG study that even faces previously paired only once with negative or positive contextual information, are rapidly processed differently in the brain, already between 30 and 60 ms post-face onset. More precisely, the faces previously seen in a positive (happy) emotional context evoked a dissociated neural response as compared to those previously seen in either a negative (angry) or a neutral context. Source localization revealed two main brain regions involved in this very early effect: the bilateral ventral, occipito-temporal, extrastriate regions and the right anterior medial temporal regions. A recent study showed in two experiments that neutral faces which were paired with negative, positive, or neutral gossip (and were then presented alone in a binocular rivalry paradigm (faces were presented to one eye, houses to the other), only the faces previously paired with negative (but not positive or neutral) gossip dominated longer in visual consciousness (Anderson et al., 2011). These findings also demonstrate that social affective learning can influence vision in a completely top-down manner, independent of the basic structural features of a face. It is important to note that the contextual influences described here are based on previous encounters, the contextual information itself is not present at the time the face is seen again. All of these findings, however, were obtained with neutral faces only and have to be extended to emotional facial expressions as well.

In addition to the conditioning literature reviewed above, recognition memory studies employing old/new paradigms have also revealed massive context effects during encoding on recognition memory. It has been shown, for example, that the N170 amplitude during recognition is diminished when the face was presented in a contextual frame compared to no contextual frame during the encoding phase, but heightened when the contextual frame was of positive compared to negative valence (Galli et al., 2006). Similar effects have been observed when fearful faces were shown at encoding, but neutral faces of the same identity at retrieval (Righi et al., 2012), which has been also shown in a recent fMRI study, where several brain regions involved in familiar face recognition, including fusiform gyrus, posterior cingulate gyrus, and amygdala, plus additional areas involved in motivational control such as caudate and anterior cingulate cortex, were differentially modulated as a function of a previous encounter (Vrticka et al., 2009). Also, attractiveness during encoding was found to alter ERP responses during retrieval (Marzi and Viggiano, 2010). Additionally, faces associated earlier with positively or negatively valenced behaviors elicited stronger activity in brain areas associated with social cognition such as paracingulate cortex and STS (Todorov et al., 2007). However, a recent study investigating the effect of concurrent visual context during encoding of faces showed that facial identity was less well recognized when the face was seen with an emotional body expression or against an emotional background scene, compared to a neutral body or a neutral background scene. Most likely, this is due to orienting responses triggered by the visual context, which may lead to a less elaborate processing and in turn resulting in a decreased facial recognition memory (Van den Stock and de Gelder, 2012). In general, context cues present at the encoding phase have a great impact on how faces are remembered.

#### Implicit race bias

An interesting example for an interaction of within-face and within-perceiver contextual features is illustrated by research investigating the impact of race bias and stereotypes on the perception of faces. Effects of implicit race bias on face perception have been demonstrated both at the neural level and at the perceptual level. For example, participants with high implicit race bias (measured using the implicit association test, IAT) showed higher increases in amygdala activation toward black faces compared to participants with lower bias (Phelps et al., 2000; Cunningham et al., 2004). Similarly, multi-voxel pattern decoding of the race of a perceived face based on BOLD signal in FFA has been shown to be restricted to participants with high race bias (Brosch et al., in press), suggesting that race bias may decrease the similarity of high-level representations of black and white faces. With regard to the face-sensitive N170 component of the ERP, it has been found that pro-white bias was associated with larger N170 responses to black versus white faces, which may indicate that people with stronger in group preferences may see out-group faces as less normative and, thus, require greater engagement of early facial encoding processes (Ofan et al., 2011). At the behavioral level, the mental templates of out-group faces possess less trustworthy features in participants with high implicit bias (Dotsch et al., 2008). Race bias furthermore has been shown to interact with the perception of emotion in a face: in a change detection task, participants observed a black or white face that slowly morphed from one expression to another (either from anger to happiness or from happiness to anger) and had to indicate the offset of the first expression. Higher implicit bias was associated with a greater readiness to perceive anger in Black as compared with White faces, as indicated by a later perceived offset (or earlier onset) of the anger expressions (Hugenberg and Bodenhausen, 2004). Similarly, positive expressions were recognized faster in White as compared with Black faces (Hugenberg, 2005).

#### Personality traits

Obviously, the personality traits of the perceiver are important when discussing to within-perceiver features. As this vast literature is worth a review in its own, we only briefly summarize findings on some personality dimensions here (for extensive reviews, see Calder et al., 2011; Fox and Zougkou, 2011). Extraversion, for example, seems to be associated with prioritized processing of positive facial expressions on neural (higher amygdala activation) and behavioral level (Canli et al., 2002), whereas higher levels of trait-anxiety and neuroticism are associated with stronger reactions to negative facial expressions, as suggested by converging evidence from different paradigms. For example, it has been shown that the left amygdala of participants reporting higher levels of state-anxiety reacts more strongly to facial expressions of fear (Bishop et al., 2004b). Moreover, a lower recruitment of brain networks involved in attentional control including the dorsolateral and ventrolateral prefrontal cortex (dlPFC, vlPFC) was observed in participants reporting increased trait-anxiety (Bishop et al., 2004a). Another study found strong associations between amygdala activity in response to backward masked fearful faces and high levels of self-reported trait-anxiety (Etkin et al., 2004). Alterations in facial expressions processing are – not surprisingly – most evident in traits related to disorders with social dysfunctions like social phobia/anxiety (e.g., Wieser et al., 2010, 2011, 2012b; McTeague et al., 2011) and autism-spectrum disorders (for a review, see Harms et al., 2010). Whereas in social anxiety biases and elevated neural responses to threatening faces (i.e., angry faces) are most commonly observed (Miskovic and Schmidt, 2012), person with autism-spectrum disorders are often less able to recognize emotion in a face (e.g., Golan et al., 2006). Furthermore, people high on the autistic spectrum seem to process faces more feature-based when asked to identify facial expressions (Ashwin et al., 2006). In addition, emerging evidence suggests that processing of facial expressions is also depending on genetic factors such as variations in the serotonin receptor gene HTR3A (Iidaka et al., 2005), the neuropeptide B7W receptor-1 gene (Watanabe et al., 2012), and serotonin transporter (5-HTT) promoter gene (Hariri et al., 2002). Noteworthy, interactions of personality traits and contextual influences are also likely to occur. A recent study demonstrated that the way how visual contextual information affected the perception of facial expressions depended on the observer’s tendency to process positive or negative information as assessed with the BIS/BAS questionnaire (Lee et al., 2012).

## Summary and Conclusion

The empirical findings reviewed in this paper clearly demonstrate that perception and evaluation of faces are influenced by the context in which these expressions occur. Basically, affective and social information gained from either within-face features (eye gaze, expression dynamics), within-sender features (body posture, prosody, affective learning), environmental features (visual scene, other faces, verbal descriptions of social situations), or within-perceiver features (affective learning, cognitive biases, personality traits) seems to dramatically influence how we perceive a facial expression. The studies as reviewed above altogether point at the notion that efficient emotion perception is not solely driven by the structural features present in a face. In addition, as the studies on affective learning and verbal descriptions of contexts show, contextual influences seem to be especially influential when the facial expression is either ambiguous (e.g., surprised faces) or no facial expression is shown (i.e., neutral faces). The latter effects not only highlight the notion of contextual influences but also pinpoint the issues of using so-called neutral faces as baseline condition in the research of facial expressions perception. Thus, using neutral faces as baseline, researchers have to be aware that these are probably influenced by the preceding faces (Russell and Fehr, 1987), which could be resolved or at least attenuated using longer inter-trial intervals, for example. Moreover, as contextual impact may be particularly high for neutral faces, one has to try to minimize contextual information when mere comparisons of neutral and other expressions are the main interest. In line with this viewpoint, it has been recently demonstrated that emotion-resembling features of neutral faces can drive their evaluation, likely due to overgeneralizations of highly adaptive perceptual processes by structural resemblance to emotional expressions (Adams et al., 2012b). This assumption is supported by a study that used an objective emotion classifier to demonstrate the relationship between a set of trait inferences and subtle resemblance to emotions in neutral faces (Said et al., 2009). Although people can categorize faces as emotionally neutral, they also vary considerably on their evaluations with regard to trait dimensions such as trustworthiness (Engell et al., 2007). A possible explanation is that neutral faces may contain structural properties that cause them to resemble faces with more accurate and ecologically relevant information such as emotional expressions (Montepare and Dobish, 2003). Another finding suggests that prototypical “neutral” faces may be evaluated as negative in some circumstances, which suggests that the inclusion of neutral faces as a baseline condition might introduce an experimental confound (Lee et al., 2008).

A large amount of the effects of context on facial expression processing seems to rely on congruency effects (i.e., facilitation of emotion perception when context information is congruent). Whereas some of the effects observed here can be explained by affective priming mechanisms (for example, when previously given verbal context influences the perception of the face presented afterward), other effects (for example, when concurrently available information from the auditory channels influences the perception of facial expressions) point to a supramodal emotion perception which rapidly integrates cues from the facial expression and affective contextual cues. Particularly the influences on surprised (ambiguous) and neutral faces show that contextual effects may play an even more important role when the emotional information is difficult to derive from the facial features alone. The latter is also in line with recent models of impression forming of other people based on minimal information (Todorov, 2011).

Beyond the scope of this paper, cultural influences obviously also define a major context for the perception of facial expressions (Viggiano and Marzi, 2010; Jack et al., 2012). In brief, facial expressions are most reliably identified by persons who share the same cultural background with senders (Ambady and Weisbuch, 2011), whereas, for instance, Eastern compared to Western observers use a culture-specific decoding strategy that is inadequate to reliably distinguish facial expressions of fear and disgust (Jack et al., 2009). Only recently it has also been shown that concurrent pain alters the processing particularly of happy facial expressions (Gerdes et al., 2012). The latter findings point at the notion that within-perceiver features also include alternations within the (central) nervous system of the perceiver.

Altogether, the studies reviewed above demonstrate that faces do not speak for themselves (Barrett et al., 2011), but are always, known or unbeknown to the perceiver, subject to contextual modulations lying within-face, within-sender, within-perceiver, or in the environment. Thus, the assumption of modular basic emotion recognition might only hold true for very rare cases, particularly when elicited emotions and resulting facial expressions are intense and exaggerated, and there is no reason to modify and manage the expression. These assumptions are also in line with the notion that even simple visual object perception is highly dependent on context (Bar, 2004).

The importance of context is congruent with several other theoretical approaches, such as Scherer’s (1992) assumption that facial expressions do not solely represent emotional, but also cognitive processes (for further discussion, see Brosch et al., 2010). In this view, facial expressions do not categorically represent few basic emotions, but are the result of sequential and cumulative stimulus evaluations (which also take into account context variables as reviewed above). Thus, facial expressions are supposed to simultaneously and dynamically express cognition and emotion. The categorical approach is further challenged by social-ecological approaches, which assume social cognition and perception to proceed in an ecologically adaptive manner (McArthur and Baron, 1983). Here it is proposed that different modalities are always combined to inform social perception. Hence, identification of facial expressions is especially fast and accurate when other modalities impart the same information. In this line, facial expressions also are contingent on the broader context of the face (i.e., what we refer to as within-face features) and the unexpressive features of the face/sender (i.e., within-sender features). This context-dependency is assumed to reflect adaption processes of humans to their ecological niche (Ambady and Weisbuch, 2011).

**Figure 2:**
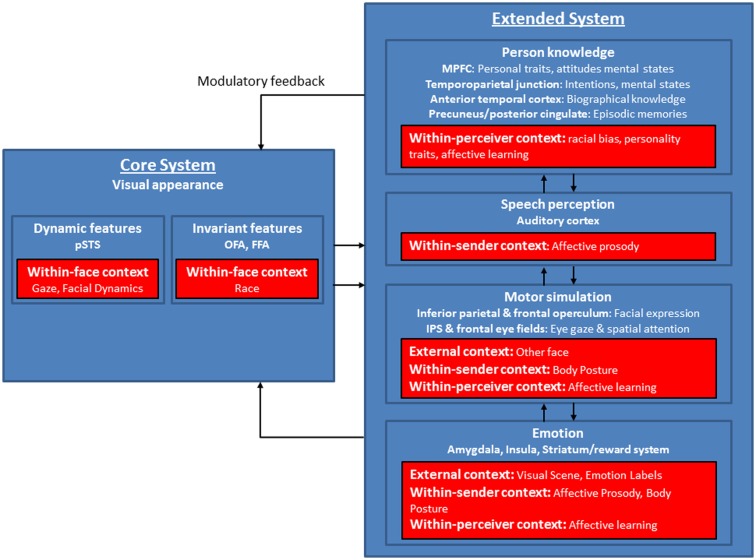
**Modified version of the model of distributed neural systems for face processing (blue boxes)**. The red boxes indicate the contextual factors as outlined in the review and show distal cues (within-face, within-sender features), influences from the transmission phase (environmental features), and proximal perceptions (within-perceiver features) and their supposed target areas within the model. Modified after Haxby et al. (2000), Haxby and Gobbini (2011).

With regard to current neurocognitive models of face processing, we suggest that the model of distributed neural systems for face perception (Haxby and Gobbini, 2011) offers an avenue for integrating the findings of different sources of context as reviewed above, as it allows for multiple entry points of context into ongoing face processing (see Figure 2).

At different levels of face processing, within-face, within-sender, within-perceiver, and external features have been shown to strongly modulate the neurocognitive mechanisms involved in face perception. The neural substrates of these modulations mainly concern brain areas within the extended network of the model (see Figure 2), but also some additional areas related to self-referential processing, impression formation, and affective learning. Context variables like within-perceiver features as learning mechanisms and personality traits influence areas involved in person knowledge and emotion processes (medial PFC, temporo-parietal junction, limbic system), which are also modulated by within-sender features like affective prosody and body posture. External features most likely modulate face processing via emotional circuits (amygdala, insula, striatum), which through feedforward and feedback loops especially between the amygdala, and primary visual cortex, OFA, FFA, and STS (Carmichael and Price, 1995; Morris et al., 1998; Iidaka et al., 2001; Amaral et al., 2003; Catani et al., 2003; Vuilleumier et al., 2004; Price, 2007), may foster the interaction between facial expressions and affective context and lead to a unified and conscious percept (Tamietto and de Gelder, 2010). Activations in the extended network in turn influence processing in the core face network via top-down modulatory feedback volleys (Haxby and Gobbini, 2011). It is important to note that the influences of context might be seen even in early stages of face processing taking place in the core system (OFA; FFA; STS). Particularly within-face features are key players here, as the STS is clearly involved in gaze processing and processing of facial dynamics.

Besides the involved structures of the brain (“where?”), it is also essential for our further understanding of contextual influences on face processing to identify at which temporal stage of the processing stream (“when?”) the integration of context and facial expression takes place. To this end, measures with high temporal resolution like EEG are much better suited than measures with high spatial, but poor temporal resolution like fMRI (Brosch and Wieser, 2011). Indeed, several ERP studies point at the notion that the integration of context and facial expression seems to be an automatic and mandatory process, which takes place very early in the processing stream, probably even before the structural encoding is completed. Looking at combinations of facial expressions and body postures, the P1 component of the ERP was enhanced when faces were presented together with an incongruent body posture (Meeren et al., 2005), which suggests a rapid neural mechanism of integration, or at least an early sensitivity for non-matching. This assumption is also supported by EEG studies on face-voice integration (de Gelder et al., 2006), where it has been found, for instance, that the auditory N1 component occurring about 110 ms after presentation of an affective voice is significantly enhanced by a congruent facial emotion (Pourtois et al., 2000). ERP studies investigating how emotionally positive or negative information at encoding influences later recognition of faces in an old/new task (Galli et al., 2006) or how concurrent visual context affects facial expression processing (Righart and de Gelder, 2006) also show effects at early encoding stages in form of a contextual modulation of the N170 amplitude. Early modifications in face-related visual cortices (STS, fusiform gyrus) may be due to re-entrant projections from the amygdala (e.g., Vuilleumier et al., 2004), crosstalk between sensory areas (bi-modal stimulation) or directly by modulations of STS activity as a hub for multisensory integration (Barraclough et al., 2005).

Altogether, the literature as reviewed above nicely fits distributed as opposed to categorical models of face processing (Haxby et al., 2000; de Gelder et al., 2003), and clearly points at the notion that the face itself might not tell us the full story about the underlying emotions. Moreover, as ERP studies reveal, the integration of context and facial expression may occur at early stages of stimulus processing and in an automatic fashion. This network is the neural substrate of a much more complex and inferential process of everyday face-reading than previously conceptualized by categorical accounts, which includes not only the perceptual processing of facial features but also social knowledge concerning context.

As this review shows, different types of context influence face perception. However, a lot of questions remain unsolved, which need to be addressed in future research. It is still unknown, for example, how these various dimensions of contexts and facial expressions are coded and how they are integrated into a single representation. Moreover, given the complexity of such a process, the available models need to be refined with regard to the interconnections between the neuroanatomical structures underlying face perception as shown in Figure 2. Particularly, the exact time course of these processes is unknown so far, and more data is needed to decide whether this is an automatic and mandatory process (de Gelder and Van den Stock, 2011). Furthermore, elucidating the interactions of different types of contexts and their influence on facial expression perception is an important research question for social neuroscience. Especially the interactions of within-perceiver and within-sender features may be of interest here, as they may enhance our understanding of non-verbal social communication. Additionally, interactions of facial expressions and contexts have so far mainly been investigated in one direction (namely the effect of the context on the face) at the perception stage. However, it would also be interesting to investigate the impact of facial expressions on how the surrounding environment is affectively toned. Moreover, the reviewed results and especially the finding of a highly flexible perception of neutral faces suggest that standard paradigms for studying facial expression perception may need some modifications. As a minimal requirement, experimenters in facial expression research need to consider the intrinsically involved contextual influences. This is all the more important as experiments on language and other faces as contexts show that even within experimentally sound studies contextual influences cannot be precluded.

Taken together, the work reviewed here demonstrates that the impact of contextual cues on facial expression recognition is remarkable. At the neural level, this interconnection is implemented in widespread interactions between distributed core and extended face processing regions. As outlined earlier, many questions about these interactions are still unsolved. All the more, the time has come for social affective neuroscience research to put faces back in context.

## Conflict of Interest Statement

The authors declare that the research was conducted in the absence of any commercial or financial relationships that could be construed as a potential conflict of interest.

## Footnotes

^1^ Moreover, the stimuli usually consist of prototypical expressions posed by models, sometimes even actors. As such, they are hardly representative, but may sometimes appear exaggerated and caricatural (Scherer, 1992).

^2^ The N170 reflects the structural encoding of faces (Bentin et al., 1996). Results on the affective modulation of the N170 are mixed so far: several studies exist which point at no modulation of the N170 components by facial expressions (e.g., Krolak-Salmon et al., 2001; Eimer and Holmes, 2002; Schupp et al., 2004; Wieser et al., 2012a), whereas others reported differences in the amplitude of the N170 between emotional and neutral faces (Batty and Taylor, 2003; Blau et al., 2007; Mühlberger et al., 2009; Wieser et al., 2010; Wronka and Walentowska, 2011). Most likely, differences are due to methodological differences between paradigms. Recently, it has been suggested that modulations of the N170 are observed only when participants attend to the facial expressions (Wronka and Walentowska, 2011).
